# Pd-Loaded Cellulose NanoSponge as a Heterogeneous Catalyst for Suzuki–Miyaura Coupling Reactions

**DOI:** 10.3390/gels8120789

**Published:** 2022-12-02

**Authors:** Laura Riva, Gloria Nicastro, Mingchong Liu, Chiara Battocchio, Carlo Punta, Alessandro Sacchetti

**Affiliations:** 1 Department of Chemistry, Materials, and Chemical Engineering “G. Natta” and INSTM Local Unit, Politecnico di Milano, 20131 Milan, Italy; 2 Department of Science, Roma Tre University, Via della Vasca Navale 79, 00146 Rome, Italy; 3 Istituto di Scienze e Tecnologie Chimiche, “Giulio Natta” (SCITEC), National Research Council-CNR, 20131 Milan, Italy

**Keywords:** nanocellulose, Suzuki–Miyaura coupling, heterogeneous catalysis, sustainable catalyst, nanocellulose-based xerogels, green chemistry

## Abstract

The (eco)design and synthesis of durable heterogeneous catalysts starting from renewable sources derived from biomass waste represents an important step for reducing environmental impacts of organic transformations. Herein, we report the efficient loading of Pd(II) ions on an eco-safe cellulose-based organic support (CNS), obtained by thermal cross-linking between TEMPO-oxidized cellulose nanofibers and branched polyethyleneimine in the presence of citric acid. A 22.7% *w*/*w* Pd-loading on CNS was determined by the ICP-OES technique, while the metal distribution on the xerogel was evidenced by SEM–EDS analysis. XPS analysis confirmed the direct chelation of Pd(II) ions by means of the high number of amino groups present in the network, so that further functionalization of the support with specific ligands was not necessary. The new composite turned to be an efficient heterogeneous pre-catalyst for promoting Suzuki–Miyaura coupling reactions between aryl halides and phenyl boronic acid in water, obtaining yields higher than 90% in 30 min, by operating in a microwave reactor at 100 °C and with just 2% *w*/*w* of CNS-Pd catalyst with respect to aryl halides (4.5‰ for Pd). At the end of first reaction cycle, Pd(II) ions on the support resulted in being reduced to Pd(0) while maintaining the same catalytic efficiency. In fact, no leaching was observed at the end of reactions, and five cycles of recycling and reusing of CNS-Pd catalyst provided excellent results in terms of yields and selectivity in the desired products.

## 1. Introduction

Pd-catalyzed Suzuki–Miyaura cross-coupling is one of the most investigated C-C bond formation reactions, widely applied for the synthesis of complex molecules, including pharmaceuticals, semiconductors, supramolecular structures, and pesticides [1,2,3,4].

Many efforts have been devoted over the years to the design and synthesis of Pd(II)-precatalysts by selecting proper ligands such as *N*-heterocyclic carbenes [5,6,7], phosphine [8,9], palladacycles [10], the PEPPSI (pyridine-enhanced precatalyst preparation) system [11], and allyl-based ligands [12], in order to improve the efficiency of this catalysis under homogeneous conditions.

Ligands guarantee excellent donor abilities, high steric hindrance, and, in most cases, the stabilization of a Pd(0) reduced form, which is considered to be the active species once generated in situ [13].

While most of these approaches allow operation at low catalyst loadings, under mild conditions, and even in green solvents, including water [14,15], they all suffer from the limits related to homogeneous catalysis, which can be summarized in the direct costs for catalysts’ synthesis, and the indirect ones for their efficient recovery and reuse, which is also a key issue for the environmental impact of the process.

For these reasons, in recent years many solutions have been proposed for the immobilization of Pd(II)-precatalysts onto heterogeneous networks [16,17,18,19], also opening the route to continuous-flow synthetic processes [20,21]. However, in most cases the pre-functionalization of solid supports with proper ligands is necessary to guarantee high efficiency in fixing Pd(II), minimizing leaching phenomena [22,23]. Moreover, the increasing demand for bio-based materials derived from renewable sources, such as biomass waste, in the framework of the circular economy, pushes towards the design of new solutions as heterogenous supports for organometallic catalysis, capable of minimizing synthetic steps and providing sustainable solutions with low environmental impact.

In this context, in recent years we designed and developed a microporous cellulose-based nanosponge (CNS), having TEMPO ((2,2,6,6-Tetramethylpiperidin-1-yl)oxyl)-oxidized cellulose nanofibers and 25 kDa branched polyethylenimine (bPEI) as main components [24]. The high porosity of the system derived from the freeze-drying process followed for converting the original hydrogel-like suspension of the two polymers into the resulting xerogel, with ice crystals acting as pores’ templates, while its chemical stability was guaranteed by the thermally induced (~100 °C) formation of amide bonds between the carboxyl groups of the oxidized nanocellulose and the amine groups of the polyamine. Further optimization of pristine formulations by addition of citric acid (CA) allowed the nanostructure to have higher mechanical resistance [25] and to better fix bPEI, improving the eco-safety [26,27,28,29] and the sustainability [30] of the material. More recently, the nanoporosity of the material was revealed by small-angle neutron scattering (SANS) investigation [31] and by FTIR-ATR analysis of the H-bond network, which evidenced water nanoconfinement in the nanostructure [32,33].

CNS have found ample application in different fields, including wastewater remediation [29,34], sensing [35,36], as drug-delivery systems [25], and as heterogenous catalysts for promoting amino-catalyzed organic reactions [37]. Very recently, we were inspired by the high heavy-metal adsorption efficiency of CNS exploited in wastewater treatment [24,29]. This property had to be ascribed to the strong chelating action of primary, secondary, and tertiary amino groups, provided by the presence of bPEI in the xerogel network. Inspired by this behavior, we envisioned the opportunity to consider these materials as suitable organic heterogeneous supports for transition metal ions, opening the synthesis of a new class of heterogeneous organometallic catalysts for organic reactions. In a first attempt, we confirmed this hypothesis by designing CNS-Cu- and CNS-Zn-loaded catalysts, which were successfully used to promote the synthesis of aromatic acetals [38].

With these premises, herein we propose CNS as easy-to-prepare biobased and sustainable supports for Pd(II) ions. As CNS do not require a pre-functionalization by specific ligands to efficiently trap Pd(II), the environmental and economic impact of these systems is minimized. The new CNS-Pd(II) composite turned out to be an efficient and stable heterogeneous precatalyst towards the Suzuki–Miyaura cross-coupling reaction.

## 2. Results and Discussion

### 2.1. CNS-Pd Synthesis and Characterization

**Scheme 1:**
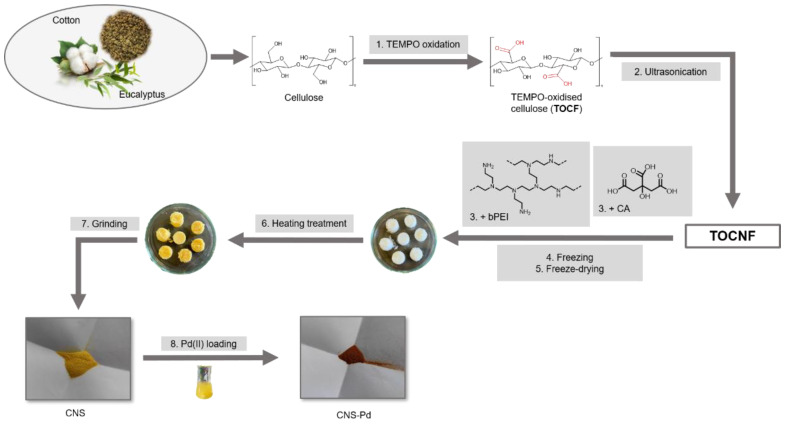
Preparation of CNS-Pd.

CNS-Pd was synthesized as reported in Scheme 1, following a two-step procedure: (i) the production of CNS and (ii) the loading of Pd(II) ions on the resulting xerogel. For the first step, TOCNF and bPEI were mixed in a 1:2 weight ratio in deionized water in the presence of 18% of CA with respect to primary amino groups of bPEI. The resulting hydrogel was transferred in molds and underwent a freeze-drying process, providing a highly porous xerogel. The latter was heated in an oven at ~100 °C in order to impart chemical stability and mechanical resistance to the final material and allowing the formation of amidic bonds by dehydration between the carboxylic groups of TOCNF and CA, and the primary amino groups of bPEI [25]. In the second step, the nanosponge was ground in a mortar before use to increase the superficial area and consequently the Pd-sorption efficiency. Pd loading was performed by soaking the CNS material in a saturated PdCl_2_ solution, running several loading cycles.

**Figure 1:**
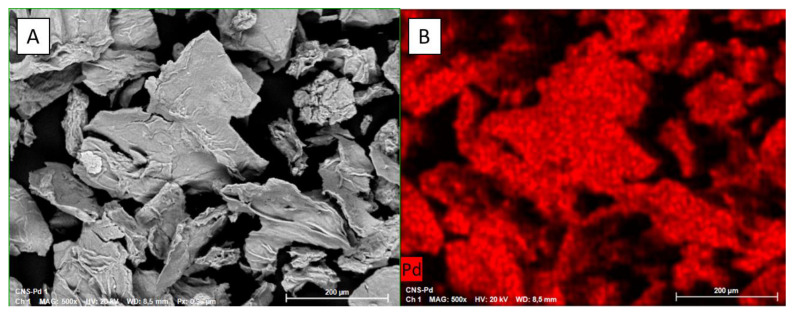
SEM images of CNS-Pd (**A**) and distribution of Pd on the catalyst’s surface, obtained with EDS (**B**).

A complete chemical and morphological characterization of CNS has already been reported in the literature. More specifically, evidence of amide bonding was shown by FTIR [32,34] and ^15^N CP-MAS solid-state NMR analyses [25]; microporosity of the system was detected by scanning electron microscopy (SEM) images and better-investigated by microcomputed tomography quantitative analysis, resulting in about 70% of the bulk material [25,29]; finally, nanoporosity of the xerogel was revealed by a small-angle neutron scattering (SANS) [31] study and an investigation of water nanoconfinement in the network by ATR-FTIR [32,34]. We thus proceeded with an in-deep characterization of the new Pd-loaded system. Scanning Electron Microscopy/Energy Dispersive Spectroscopy (SEM–EDS) analysis revealed a homogeneous distribution of the metal on ground CNS (Figure 1).

**Figure 2:**
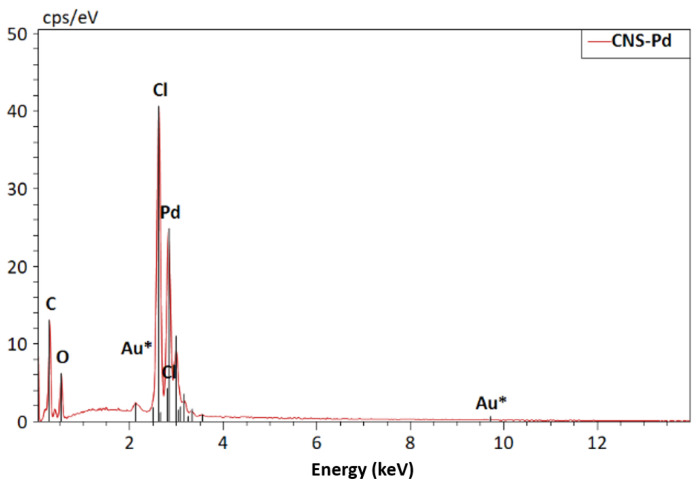
EDS absorption spectrum of CNS-Pd.

An EDS absorption spectrum is also reported in Figure 2, where the Pd and Cl^−^ signals can be clearly observed.

A quantification of the amount of Pd(II) actually loaded on CNS-Pd was measured by ICP-OES analysis, obtaining a value of 22.7% *w*/*w*, corresponding to 2.13 mmol_Pd_/g_CNS_.

To obtain information on the structural features of the CNS-Pd catalyst, X-ray photoelectron spectroscopy (XPS) studies were performed on the pristine catalyst as well as on the catalyst recovered after the first catalytic cycle (see Section 2.6 for reaction conditions). The pristine CNS matrix was also investigated to provide useful references for the assignment of Pd3d spectral features. Measurements were carried out at C 1s, N 1s, O 1s, and Pd 3d core levels. A complete collection of core-level binding energy (BE), full width at half-maxima (FWHM) values, and proposed assignments is reported in Table S1 in the Supporting Information; here, the Pd3d spin-orbit components will be discussed with particular attention since they are of major interest for the assessment of the Pd–CNS interaction in the catalyst and for the investigation of its role in the catalytic reaction. C1s and N1s spectral features will also be briefly discussed since the reproducibility of such signals confirms the stability of the catalyst molecular structure upon use.

**Figure 3:**
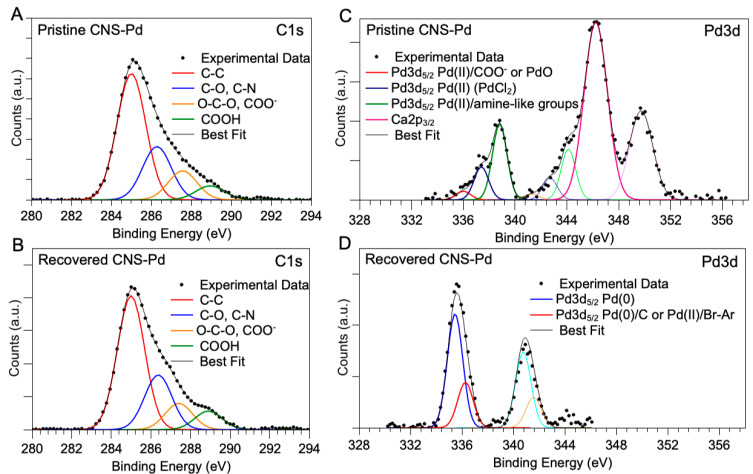
(**A**) XPS C 1s core-level spectra acquired on pristine CNS and (**B**) CNS recovered after catalysis; (**C**) XPS Pd 3d core-level spectra acquired on CNS before and (**D**) after catalysis.

C1s and N1s spectra collected on the pristine CNS and on the CNS-Pd catalyst before and after the catalytic process show analogous features, in excellent accordance with the chemical composition of CNS; in more detail, C1s spectra are composite and at least four spectral components can be individuated by applying a peak fitting procedure, assigned, respectively, to aliphatic C atoms (BE = 285.00 eV), C atoms bonded to N or O in C-N, C-O functional groups (286.3 eV), O-C-O or C=O carbons (287.5 eV), and carboxylic COOH functional groups (288.9 eV) [39,40,41,42,43]. The relative amount of each species is well-reproducible in the three samples, as reported in the column “atomic ratios” in Table S1. C1s spectra of CNS-Pd pristine and recovered from the catalysis reaction are reported in Figure 3A,B. As for the N 1s spectra, a main signal is always found at about 400 eV, as expected for N atoms in the polyamine [43]. At higher BE values, a signal of very low intensity is also observed, probably due to oxidized nitrogen atoms belonging to impurities of the CNS.

Pd3d spectra show the presence of several contributions in different BE positions for the pristine and recovered catalysts, as reported in Figure 3C (pristine CNS-Pd) and D (recovered CNS-Pd).

The Pd3d spectrum of pristine CNS-Pd shows the presence of Pd(II) ions only; however, by applying the peak-fitting procedure, it is possible to point out at least three spin-orbit pairs whose BE positions are indicative of different chemical environments. The signal at lower BE (Pd3d_5/2_ BE = 337.38 eV) can be associated with PdO [43] or, as suggested by some authors, with Pd(II) ions coordinating carboxylic groups [44]. A less-suitable assignment could be atomic Pd(0) interacting with amorphous carbon, as suggested by Bertolini et al. [45]. Moreover, the low-BE component is also less intense (about 7% of all Pd contribution) in the pristine CNS-Pd Pd3d spectrum. The second feature (Pd3d_5/2_ component at 338.77 eV BE) is indicative of ionic Pd(II) in PdCl_2_ [43], as also confirmed by the position of the Cl2p signal (Cl2p_3/2_ BE = 198.40 eV, see Table S1) [46]. Finally, the most intense signal, at higher BE value (Pd3d_5/2_ BE = 340.19 eV, 64% of all the palladium in the catalyst), is due to Pd(II) ions interacting with the amine-like nitrogens of the polyamine moieties in coordination compounds, similarly to Pd(NH_3_)_4_Cl_2_ [43].

In Figure 3C, a Ca2p signal arising from Ca(II) ions contained as impurities in the CNS matrix is observed and partially superimposed to the 3/2 Pd3d spin-orbit components; an analogous signal is also observed in pure-pristine CNS (see Supporting Information, Table S1). The Ca2p signal is not found in the recovered catalyst, probably due to repeated washing with ultrapure water.

After recovering, the Pd signal measured for CNS-Pd catalyst suggests a different composition. The most-intense spectral component (71% of Pd species) is shifted at low BE values (Pd3d_5/2_ BE = 335.46 eV) and it is now indicative of reduced Pd(0) atoms. At higher BE values, a less intense signal can be observed (Pd3d_5/2_ BE = 338.20); from the literature, this minor (29%) contribution could be due to Pd(0) atoms interacting with an amorphous carbon matrix [45] or Pd(II) ions coordinating halogenated organic molecules (Br-containing reactant residues, for example, or reaction intermediates and byproducts) [47,48]. Measurements at the Br3d core level reveal a small number of bromine atoms covalently bonded to C (Br3d_5/2_ BE = 68.80 eV) in the recovered sample, supporting the latter assignment (see Table S1).

### 2.2. Suzuki–Miyaura Reaction Optimization

**Scheme 2:**
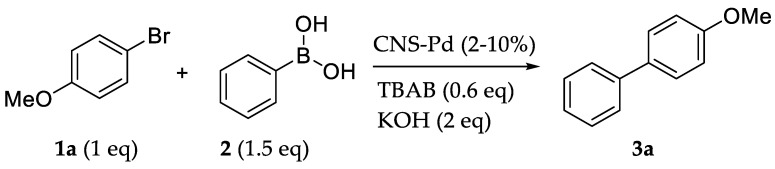
Suzuki–Miyaura reference reaction.

The reaction between 4-Br-anisole (**1a**) and phenylboronic acid (**2**) in water (Scheme 2) was selected as the model for the optimization of the conditions. CNS-Pd precatalyst was used in 2–10% *w*/*w* with KOH (2 eq) as base. In addition, to facilitate the solubilization of the reagents, the phase-transfer agent TBAB (0.6 eq) was used. ^1^H-NMR in CDCl_3_ with acetonitrile as internal standard was used to calculate the reaction conversion during the reaction optimization process. In all cases, a complete selectivity toward the desired product could be observed, without the formation of any undesired by-products.

The influence of reaction time and temperature was first investigated with the use of a microwave reactor, according to the principle of “time–temperature equivalence”, shortening the reaction time under the condition of increasing temperature. For comparison, conventional heating was also considered.

As reported in Table 1, after working at room temperature (T = 25 °C) with long reaction time (48 h), the reaction yield was not satisfactory (entry 1). As expected, at higher temperatures yields increased and shorter reaction times could be applied, going from 80 °C, 30 min (entry 2), with a yield of 57% to 100 °C, 20 min (entry 3), with a yield of 72%. Finally, applying a temperature of 100 °C for a reaction time of 30 min (entry 4), a satisfying 92% yield was achieved. It has to be noticed that in the same conditions but with the use of conventional heating, a very low 19% yield was obtained (entry 5), thus highlighting the unique role and the high effectiveness of microwave irradiation in this catalytic system.

In order to achieve higher yields, the amount of catalyst was increased (Table 2). No significant results were obtained. Indeed, going from 2% (entry 2) to 5% (entry 3) and 10% *w*/*w* (entry 4), the yields did not substantially improve. As expected, in the absence of catalyst (entry 1) and in the presence of a metal-free catalyst (CNS, entry 5), no conversion was observed. According to these results, the 2% *w*/*w* catalyst loading was taken as the optimal condition. It is important to highlight that according to the measured loading of palladium on CNS, the 2% *w*/*w* of catalyst corresponds to a very low 0.45% *w*/*w* amount of Pd, making this system very efficient in terms of metal loading in the reaction.

The role of the base was also considered, and different inorganic bases, namely, NaOAc, Na_2_CO_3_, K_2_CO_3_, and KOH, were tested in these conditions. Results are reported in Table 3.

The results obtained show that, in base-free conditions (entry 1), the pH is probably not alkaline enough to promote the reduction of Pd (II) to Pd (0), thus obtaining poor yields [49]. With the use of NaOAc, Na_2_CO_3_, or K_2_CO_3_ (entry 2, 3, and 4), an adequate alkaline environment could not be provided, with a consequent unsatisfactory activation of the catalytic cycle, resulting again in low yields [49]. The only base that gave good results was the strong base KOH, able to give a yield of 92% (entry 5).

Finally, the use of the phase-transfer agent was explored. As the coupling reaction is run in water and under heterogeneous conditions, producing water-insoluble products from organic reagents, tetrabutylammonium bromide (TBAB) was added to the reaction mixture. Optimization of the amount of TBAB in the reaction was performed by gradually reducing it from the starting 0.6 to 0.15 equivalents, obtaining the results shown in Table 4.

The results underline that the reaction yield was somewhat higher when using small amounts of TBAB (97% with 0.15 eq, entry 3). It can be also noticed that, without using TBAB, acceptable yields can be obtained (90%, entry 4). The role of TBAB turned out to be crucial when scaling up the reaction. In fact, when performing a ×10 reaction scale-up (entry 5 and 6), the presence of TBAB was essential in increasing the efficiency of the reaction in the presence of large quantities of polar solvent. In this case, without the use of TBAB, a low 60% yield was indeed obtained.

After all this screening, the optimized conditions were defined as follows: 100 °C, 30 min, 2% *w*/*w* catalyst, corresponding to 0.45% *w*/*w* of palladium, KOH as base, and 0.15 equivalents of TBAB. For the model Suzuki–Miyaura reaction, in these conditions a TON = 1.2 × 10^2^ and a TOF = 6.5 × 10^−2^ s^−1^ could be calculated. These values are reasonably good and comparable to many industrial catalytic processes, but are obtained by operating in the presence of an eco-friendly cellulose-derived palladium support. In fact, while we are aware that higher TONs can be obtained under homogeneous conditions, these values are comparable or even better than those of most of the examples reported in the literature for fixing Pd on heterogeneous supports [16,17,18,20,21,22,23]. Moreover, in this case, the choice of the support falls on a bio-based system derived from waste biomass.

To verify the reliability of this catalytic system, a complete mass recovery of the reaction after column chromatography purification was performed. Reaction conditions, work-up, and purification are described in detail in the Section 4 and with this test we were able to verify that, in the optimized conditions after the purification process, a 95% yield could be achieved, calculated by weighting the white crystalline powder obtained after the whole process.

### 2.3. Suzuki–Miyaura Substrate Scope

After the definition of the optimized conditions, different substrates were tested in the reaction. All the experiments are reported in Table 5.

An early analysis on the position of the methoxyl substituent was carried out. As can be seen from the results (entry 1, 2, and 3), with the optimized reaction conditions it was possible to obtain excellent yields with the substituent in para- and meta-position. In the case of the methoxy substituent in the ortho- position (entry 3), we observed a decrease in yield, probably due to the steric effect of the substituent in the position adjacent to the halogen involved in the reaction mechanism. The test with more catalyst (10% *w*/*w*, entry 4) further confirmed the low conversion, supporting the hypothesis of the steric effect of the substituent.

After the position analysis, a study with structurally different substituents was then performed. In the absence of substituents on the benzene ring of the organ halide (entry 8), a satisfactory yield of more than 90% was obtained. In the presence of electron-donating activating substituents on the benzene ring in para-position, such as -CH_3_ (entry 5) and -NH_2_ (entry 9), good yields were achieved, in particular a yield of 72% with p-Br-toluene and 76% with p-Br-aniline. In the presence of an electron-withdrawing group, such as -CHO (entry 6), a dramatic drop in reaction efficiency could be observed, reaching a yield around only 13%. When the reaction was conducted increasing the catalyst amount to 10% *w*/*w*, we noticed an increase in the yield, reaching a good value (80%, entry 7), finding that the reduced reactivity of the starting aril-halide could be balanced by increasing the amount of catalyst, thus achieving good conversions and maintaining selectivity toward the desired product. In contrast, surprisingly, the presence of the electron-withdrawing group -CN in the ortho-position (entry 10) gave an extremely high yield (99%), comparable with the yield obtained with p-Br-anisole (entry 1). By changing the halogen involved in the coupling reaction and considering Cl-benzene (entry 11) instead of Br-benzene (entry 8), we observed a decrease in yield from 93% to 55.5%. Once again, these values are in line with those reported for several heterogeneous supports [16,17,18,20,21,22,23].

### 2.4. Multiple Cross-Coupling Suzuki–Miyaura Reactions

**Scheme 3:**
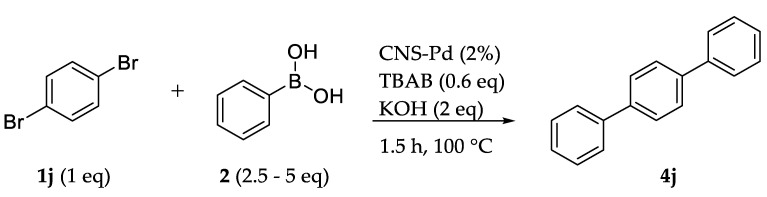
Reaction of double cross-coupling with 1,4-dibromobenzene.

**Scheme 4:**
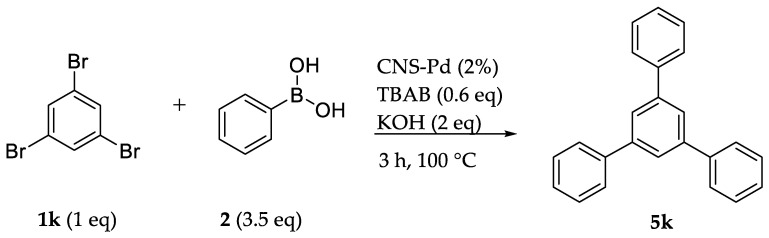
Reaction of triple cross-coupling with 1,3,5-tribromobenzene.

After studying the effect of substituents, we then performed tests to confirm that CNS-Pd could also be used for multiple couplings. For this study, we selected 1,4-dibromobenzene (Scheme 3) and 1,3,5-tribromobenzene (Scheme 4) as organ halides, increasing the amount of phenylboronic acid (2.5 equivalents for **1j** and 3.5 equivalents for **1k**) and slightly changing the reaction conditions (reaction time increased to 1.5 h for **1j** and to 3 h for **1k**).

In these reaction conditions, we were able to obtain high selectivity (>95%) towards the product **4j** in the reaction with 1,4-dibromobenzene (Scheme 3), reaching a conversion of 55%. Increasing the phenylboronic acid amount (from 2.5 to 5 equivalents compared to **1j**) and maintaining the same reaction conditions (1.5 h, 30 °C), we were able to reach a very high conversion (90%) without losing selectivity toward product **4j**.

In the reaction with 1,3,5-tribromobenzene (Scheme 4), under the conditions of 3 h, at 100 °C, with 3.5 equivalents of **2** compared with **1k** reagent, we obtained a very high selectivity (>95%) towards product **5k**, with a conversion of 82%, confirming the catalytic efficiency of CNS-Pd also in multiple cross-coupling reactions.

### 2.5. Leaching Tests

Tests were performed to evaluate the leaching of Pd from the CNS-Pd catalyst under the reaction conditions (see Section 4). The amount of Pd in solution before and after the reaction was evaluated by means of ICP-OES analysis. After one reaction cycle, only 7% of the initial amount of metal (0.016 mg when 1 mg of CNS-Pd is used) was released in the solution. No significant metal loss was observed after a second cycle, thus confirming that, after stabilization, Pd remains anchored on the heterogeneous support, paving the way for the possible recycling of CNS-Pd.

### 2.6. Recyclability Tests

To evaluate the possibility of reusing CNS-Pd several times without losing catalytic activity, reusability tests were performed between **1a** and **2** to give **3a** under the optimized conditions. Five consecutive reaction runs were realized and, after each cycle, the reaction yield was evaluated by ^1^H-NMR with acetonitrile as internal standard to evaluate the efficiency of the reused catalytic system. Results are reported in Table 6.

As can be observed, CNS-Pd can be considered a heterogeneous catalyst with good reusability characteristics, as, after five cycles, more than 96% conversion was still achieved without losing selectivity towards product **3a**, demonstrating the long catalyst life.

## 3. Conclusions

In this work, cellulose-based nanosponges (CNS) were prepared. TEMPO-oxidized cellulose nanofibers and branched polyethyleneimine were cross-linked together in the presence of citric acid to obtain a micro- and nanoporous structure and this latter was then loaded with Pd(II) to obtain a potential heterogeneous catalyst for Suzuki–Miyaura coupling reactions. The morphology and structure of the material were characterized by various SEM–EDS and ICP-OES analyses. The CNS-Pd system was also thoroughly investigated by XPS analysis to evaluate the behavior of the metal in the catalytic activity. An optimization study on the heterogeneous reaction was conducted, reaching optimal condition for the obtainment of high-rate yields. The catalytic recycling ability of the material and the catalytic effect for different substituents were also examined, confirming the possibility of reusing this sustainable catalyst several times.

## 4. Materials and Methods

All of the reagents were purchased from Merck (Darmstadt, Germany). Cotton linter was obtained from Bartoli paper factory (Capannori, Lucca, Italy). Deionized water was produced within the laboratories with a Millipore Elix^®^ Deionizer with Progard^®^ S2 ion exchange resins (Merck KGaA, Darmstadt, Germany). All 1H-NMR spectra were recorded on a 400 MHz Brüker (Billerica, MA, USA) NMR spectrometer. Microwave reactions were conducted in a Biotage^®^ Initiator+ (Uppsala, Sweden). Other equipment used in the procedures includes a Branson SFX250 Sonicator (Emerson Electric Co., Ferguson, MI, USA), a SP Scientific BenchTop Pro Lyophilizer (SP INDUSTRIES, 935 Mearns Road, Warminster, UK), a Büchi Rotavapor^®^ R-124 8 (Flawil, Switzerland), and a Thermotest Mazzali laboratory oven (Monza, Italy). Scanning electron microscopy (SEM) was performed using a variable pressure instrument (SEM Cambridge Stereoscan 360) at 100/120 pA with a detector BSD. The operating voltage was 15 kV with an electron beam current intensity of 100 pA. The focal distance was 9 mm. The EDS analysis was performed using a Bruker Quantax 200 6/30 instrument (Billerica, MA, USA). The metal concentrations were measured by ICPOES atomic emission spectroscopy using a Perkin Elmer Optima 3000 SD spectrometer (Wellesley, MA, USA).

### 4.1. TEMPO-Oxidized Cellulose (TOC) Production and Titration and Synthesis of Cellulose NanoSponges (CNS)

TEMPO-Oxidized Cellulose (TOC) production and titration were performed according to a procedure previously reported in the literature [34,50,51]. After TOC synthesis and characterization, TEMPO-Oxidized Cellulose Nanofibers (TOCNF) for the production of CNS were produced by suspending 3.5 g of TOC in 0.14 L of deionized water, adding 0.210 g of NaOH pellets. The suspension was ultrasonicated with a Branson SFX250 Sonicator, achieving a homogeneous suspension of TOCNF. This latter was then acidified with 12 N HCl (2 mL), filtered under vacuum on a Büchner funnel, and washed with deionized water (250 mL) reaching neutral pH. Then, two aqueous solutions of anhydrous citric acid (CA) (1.792 g in 10 mL) and 25 kDa branched polyethylenimine (bPEI) (7 g of bPEI in 10 mL) were mixed with the TOCNF solution, while continuously stirring until reaching a white and homogeneous hydrogel and obtaining a final TOCNF concentration of 3% *w*/*w*. The mixture was then placed in well plates used as molds, quickly frozen at −20 °C, frozen-dried for 48 h at −52 °C, 140 μbar using an SP Scientific BenchTop Pro Lyophilizer, and finally thermally treated in the laboratory oven performing a heating ramp from a temperature of 55 °C to a maximum of 98 °C. This temperature was kept for 16 h. At the end of the process, CNS was ground with a mortar and then washed with deionized water (500 mL) to remove the excess bPEI. This procedure for the synthesis of CNS was described in our previous works [37,38].

### 4.2. Preparation of the Catalyst

After washing, 275 mg of CNS was ground into a fine powder. In the meantime, a saturated acidic solution of PdCl_2_ (101 mg in 50 mL of 0.1 M HCl) was prepared. Ground CNS was soaked in this solution until complete adsorption of Pd (10 min), repeating the adsorption cycle three times in the same conditions. After every sorption cycle, Pd-loaded CNS was filtered on a Buchner funnel, washed with 100 mL of deionized water and 50 mL of ethanol, and, finally, left to dry in open air, obtaining the catalyst CNS-Pd.

### 4.3. Catalyst Characterization

Scanning Electron Microscopy (SEM) was performed using a variable-pressure instrument (SEM Cambridge Stereoscan 360) at 100/120 Pa with a detector VPSE. The operating voltage was 20 kV with an electron beam current intensity of 150 pA. The focal distance was 8 mm. The specimens were analyzed in High Vacuum mode after metallization.

EDS analysis was performed using a Bruker Quantax 200 6/30 instrument.

Inductively Coupled Plasma Optical Emission Spectroscopy (ICP-OES) analysis was performed on solid materials to determine the percentage of palladium in CNS-Pd. ICP-OES was conducted using a Perkin Elmer Optima 3000 SD spectrometer and the samples were treated with nitric acid (HNO_3_) to completely dissolve the organic portion of the material before the determination of the metal concentration.

X-ray photoelectron spectroscopy (XPS) measurements were carried out using a custom designed spectrometer, described in previous studies [52] and equipped with a non-monochromatized Mg Ka X-ray source (1253.6 eV pass energy 25 eV, step 0.1 eV). For this experiment, photoelectrons emitted by C1s, O1s, N1s, Pd3d, Cl2p, Br3d, and B1s core levels were detected on powder samples of the pristine and recovered CNS-Pd catalyzer. All spectra were energy-referenced to the C 1s signal of aliphatic C atoms at 285.00 eV binding energy (BE) [53]. Atomic ratios were calculated from peak intensities using Scofield’s cross-section values [54]. Curve-fitting analysis was performed using Gaussian profiles as fitting functions after subtraction of a polynomial background. For qualitative data, the BE values were mostly referred to from the NIST database [43].

### 4.4. Suzuki–Miyaura Reaction

In a 5 mL microwave vial, CNS-Pd (1–5 mg, 2–10% *w*/*w*), KOH (0.536 mmol, 2 eq), phenylboronic acid (0.402 mmol, 1.5 eq), and 2.5 mL of water as solvent were added. Then, tetrabutylammonium bromide (TBAB) (0.0402–0.1608 mmol, 0.15–0.6 eq) and reagent **1a**–**k** (0.268 mmol, 1 eq) were put in the vial (all the percentages and equivalents are referred to as reagents **1a**–**k**). The reaction was performed under microwave irradiation in a temperature range of 40–130 °C and for reaction times between 10 min and 3 h.

### 4.5. Reaction Work-Up and Product Purification

At the end of the reaction, 2 mL of ethyl acetate was added into the reaction mixture and stirred for 10 min. The solution was then filtered in a glass straw equipped with cotton to remove the catalyst and the reaction vial was washed three times with 2 mL of water and three times with 2 mL of ethyl acetate. The organic and aqueous phases were transferred in an extractor funnel and 5 mL of 0.1 N HCl solution was added. The aqueous phase was then extracted three times with 15 mL of ethyl acetate and all the organic phases were collected together, and then washed once with 10 mL of NaOH 0.1 N and twice with 10 mL of a saturated solution of NaCl. Then, the organic phase was collected and anhydrified with Na_2_SO_4_. Lastly, the final organic solution was then filtered off and the solvent was removed under vacuum to obtain a crude for the NMR analysis. The purification of the product was performed with flash column chromatography using a solvent mixture of hexane and ethyl acetate in a 95:5 ratio.

Yields were calculated by means of ^1^H-NMR analysis using acetonitrile (ACN) as internal standard. The analysis was performed on the crude reaction without purification process. For the standard reaction (4-Br-anisole as reagent **A**), the yield calculated by internal standard technique with the ^1^H-NMR was further confirmed with the yield obtained with mass recovery after purification with column chromatography, as described in the previous paragraph.

### 4.6. Leaching Test

In a 20 mL microwave vial, CNS-Pd (12 mg), KOH (360 mg), and 15 mL of water as solvent were added. Then, TBAB (312 mg) was put in the vial. The reaction was performed under microwave irradiation at 100 °C for 30 min. Once finished, the reaction was filtered off on a Buchner funnel to remove the solid and the solution was analyzed through ICP-OES analysis to quantify the Pd released in the reaction environment.

### 4.7. Reusability Test

Re-usability tests were conducted on the reference reaction with *p*-Br-anisole (Scheme 2). In a 5 mL microwave vial, CNS-Pd (10 mg, 10% *w*/*w*), KOH (1.072 mmol, 2 eq), phenylboronic acid (98 mg), and 5 mL of water as solvent were added. Then, TBAB (0.804 mmol, 1.5 eq) and *p*-Br-anisole (0.536 mmol, 1 eq) were put in the vial. The reaction was performed under microwave irradiation at 100 °C for 30 min. At the end of the reaction time, the vial was centrifuged, and the reaction solvent was removed. Three cycles of washing of CNS-Pd inside the vial were then carried out as described: a 5 mL measure of AcOEt was added into the reaction vial, and the solution was left stirring for 10 min and subsequently centrifuged. The supernatant was removed, taking care not to remove the catalyst, and the washing was repeated twice more. At the end of the washing passages, CNS-Pd was allowed to dry inside the vial, and this latter was then used for a new reaction cycle. This procedure was repeated five times.

### 4.8. NMR Product Characterization

**Internal Standard (ACN):** ^1^H NMR (400 MHz, CDCl_3_) δ 2.04 (s, 3H); **Product 3a (4-Methoxybiphenyl):** ^1^H NMR (400 MHz, CDCl_3_) δ 7.55–7.50 (m, 4H), 7.40 (t, *J* = 7.5, 2H), 7.29 (t, *J* = 7.3, 1H), 6.97 (d, *J* = 8.8, 2H), 3.84 (s, 3H) [55]; **Product 3b (3-Methoxybiphenyl):** ^1^H NMR (400 MHz, CDCl_3_) δ 7.61 (d, *J* = 7.5, 2H), 7.45 (t, *J* = 7.6, 2H), 7.39–7.35 (m, 2H), 7.20 (d, *J* = 7.7, 1H), 7.15 (t, *J* = 2.0, 1H), 6.92 (dd, *J* = 8.2, 2.3, 1H), 3.87 (s, 3H) [56]; **Product 3c (2-Methoxybiphenyl):** ^1^H NMR (400 MHz, CDCl_3_) δ 7.54 (d, *J* = 8.3, 2H), 7.41 (t, *J* = 7.6, 2H), 7.34–7.31 (m, 3H), 7.03 (t, *J* = 7.5, 1H), 6.99 (d, *J* = 8.5, 1H), 3.81 (s, 3H) [56]; **Product 3d (4-Methylbiphenyl):** ^1^H NMR (400 MHz, CDCl_3_): δ = 7.64 (d, *J* = 8.0 Hz, 2H), 7.54 (d, *J* = 8.0 Hz, 2H),7.47 (t, *J* = 7.2, 2H), 7.37 (t, *J* = 7.2 Hz, 1H), 7.28 (d, *J* = 8.0 Hz, 2H), 2.44 (s, 3H) [55]; **Product 3e (Biphenyl-4-carbaldehyde):** ^1^H NMR (400 MHz, CDCl_3_): δ = 9.96 (s, 1H), 7.86 (d, *J* = 8.0 Hz, 2H), 7.66 (d, *J* = 8.0 Hz, 2H), 7.55 (d, *J* = 8.4 Hz, 2H), 7.40 (t, *J* = 7.2 Hz, 2H), 7.34 (t, *J* = 7.2 Hz, 1H) [55]; **Product 3f (Biphenyl):** ^1^H NMR (400 MHz, CDCl_3_): δ = 7.54 (d, *J* = 8.0 Hz, 4H), 7.39 (t, *J* = 8.0 Hz, 4H), 7.31–7.27 (m, 2H) [55]; **Product 3g (4-Aminobiphenyl):** ^1^H NMR (400 MHz, CDCl_3_, TMS) δ 7.52 (d, *J* = 7.9 Hz, 2H, CH), 7.41–7.36 (m, 4H, CH), 7.25 (t, *J* = 7.3 Hz, 1H, CH), 6.73 (d, *J* = 8.5 Hz, 2H, CH), 3.68 (s, 2H) [57]; **Product 3h (2-Cynobiphenyl):** ^1^H NMR (400 MHz, CDCl_3_): δ (ppm) 7.76 (dd, *J* = 8.0Hz, 1.2Hz, 1H), 7.64 (td, *J* = 7.6 Hz, 1.2Hz, 1H), 7.58–7.54 (m, 2H), 7.54–7.40 (m, 5H) [58]; **Product 4j (1,1′:4′,1″-terphenyl):** ^1^H NMR (400 MHz, Chloroform-d) δ 7.70 (s, 4H), 7.69–7.64 (m, 4H), 7.50–7.45 (m, 4H), 7.41–7.35 (m, 2H) ppm [43]; **Product 5k (1,3,5-Triphenylbenzene):** ^1^H NMR (400 MHz, CDCl_3_): δ = 7.71 (s, 3H), 7.63 (d, *J* = 7.2 Hz, 6H), 7.42 (t, *J* = 8.0 Hz, 6H), 7.31 (t, *J* = 6.8 Hz, 3H) [59].

## Figures and Tables

**Table 1:** Time and temperature reaction optimization ^a^.

Entry	T (°C)	T (h)	Yield (%)
1	25 (RT)	48	19
2	80	0.5	57
3	100	0.33	72
4	100	0.5	92
5 ^b^	100	0.5	19

^a^ Reaction conditions: 0.268 mmol of **1a** (1 eq), 0.161 mmol of TBAB (0.6 eq), 0.563 mmol of KOH (2 eq), and 1 mg (2% *w*/*w*) catalyst in 2.5 mL of water under MW irradiation. ^b^ Conventional heating instead of MW irradiation was used.

**Table 2:** Catalyst loading optimization ^a^.

Entry	CNS-Pd (%)	Pd (%)	Yield (%)
1	0	0.00	0
2	2	0.45	92
3	5	1.14	95
4	10	2.27	94
5 ^b^	10	-	0

^a^ Reaction conditions: 0.268 mmol of **1a** (1 eq), 0.161 mmol of TBAB (0.6 eq), 0.563 mmol of KOH (2 eq), and the listed amount of catalyst in 2.5 mL of water under MW irradiation (T = 100 °C, 30 min). The *w*/*w* percentage of catalyst is referred to as **1a**. ^b^ metal free CNS was used.

**Table 3:** Role of the base in the reaction ^a^.

Entry	Base	Yield (%)
1	-	15
2	NaOAc	54
3	Na_2_CO_3_	25
4	K_2_CO_3_	25
5	KOH	92

^a^ Reaction conditions: 0.268 mmol of **1a** (1 eq), 0.161 mmol of TBAB (0.6 eq), 0.563 mmol (2 eq) of base, and 2% *w*/*w* of catalyst in 2.5 mL of water under MW irradiation (T = 100 °C, 30 min).

**Table 4:** Role of the phase-transfer catalyst in the reaction ^a^.

Entry	TBAB Equivalents	Conversion (%)
1	0.60	92
2	0.30	96
3	0.15	97
4	0	90
5 ^b^	0	60
6 ^b^	0.15	95

^a^ Reaction conditions: 0.268 mmol of 1a (1 eq), TBAB, 0.563 mmol (2 eq) of KOH, and 2% *w*/*w* of catalyst in 2.5 mL of water under MW irradiation (T = 100 °C, 30 min). ^b^ A 10× scale-up of the reference reaction.

**Table 5:**
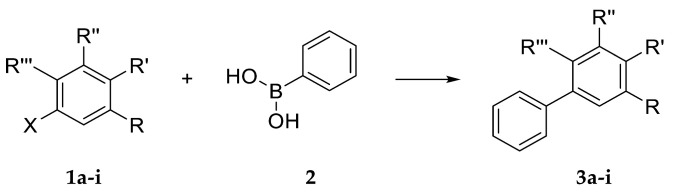
Suzuki–Miyaura cross-couplings of ArX and PhB(OH)_2_ catalyzed by CNS-Pd ^a^.

Entry	ArX	R	R′	R″	R‴	X	Conversion (%)
1	**1a**	-H	-OCH_3_	-H	-H	-Br	99
2	**1b**	-H	-H	-OCH_3_	-H	-Br	98
3	**1c**	-H	-H	-H	-OCH_3_	-Br	54
4 ^c^	**1c**	-H	-H	-H	-OCH_3_	-Br	38
5 ^b^	**1d**	-H	-CH_3_	-H	-H	-Br	72
6 ^b^	**1e**	-H	-CHO	-H	-H	-Br	13
7 ^b,c^	**1e**	-H	-CHO	-H	-H	-Br	80
8	**1f**	-H	-H	-H	-H	-Br	93
9	**1g**	-H	-NH_2_	-H	-H	-Br	76
10	**1h**	-CN	-H	-H	-H	-Br	99
11	**1i**	-H	-H	-H	-H	-Cl	55

^a^ Reaction conditions: 0.268 mmol of **1a** (1 eq), TBAB (0.15 eq), 0.563 mmol (2 eq) of KOH, and 2% *w*/*w* of catalyst in 2.5 mL of water under MW irradiation (T = 100 °C, 30 min). ^b^ A 0.6 eq value of TBAB. ^c^ A 10% *w*/*w* value of catalyst.

**Table 6:** Results from Recyclability tests ^a^.

Entry	Cycle Number	Yield (%)
1	I	97
2	II	99
3	III	98
4	IV	99
5	V	96

^a^ Reaction conditions: 0.268 mmol of **1a** (1 eq), TBAB (0.15 eq), 0.563 mmol (2 eq) of KOH, and 2% *w*/*w* of catalyst in 2.5 mL of water under MW irradiation (T = 100 °C, 30 min).

## Supplementary Materials

The following supporting information can be downloaded at: https://www.mdpi.com/article/10.3390/gels8120789/s1. Table S1: XPS data analysis results: core-level binding energy (BE), full width at half-maxima (FWHM) values, internal atomic ratios, and proposed assignments. Reference [60] is cited in the supplementary materials.
