# Supplementary material for: Pd-Loaded Cellulose NanoSponge as a Heterogeneous Catalyst for Suzuki–Miyaura Coupling Reactions
Source: Gels. 2022 Dec 2;8(12):789. doi: 10.3390/gels8120789 (PMC9778444; doi:10.3390/gels8120789)
Supplement: Supplementary file 1 [file gels-08-00789-s001.zip › gels-2059987-supplementary.pdf]

Supporting information for

Pd-Loaded Cellulose NanoSponge as Heterogeneous Catalyst for Suzuki-Miyaura Coupling Reactions

Laura Riva[1], Gloria Nicastro[1], Mingchong Liu[1], Chiara Battocchio[2], Carlo Punta[1,3], Alessandro Sacchetti[1*]

[1] Department of Chemistry, Materials, and Chemical Engineering "G. Natta" and INSTM Local Unit, Politecnico di Milano, 20131 Milano, Italy;

[2] Department of Science, Roma Tre University of Rome, Via della Vasca Navale 79, 00146 Rome, Italy;

[3] Istituto di Scienze e Tecnologie Chimiche, "Giulio Natta" (SCITEC), National Research Council-CNR, I-20131 Milan, Italy

Table S1. XPS data analysis results: core level binding energy (BE), full width at half-maxima (FWHM) values, internal atomic ratios, and proposed assignments.

Sample	Signal	BE (eV)	FWHM (eV)	Atomic Ratios	Assignment
pristine CNS	C1s	285.00	1.91	23.4	C-C
		286.42	1.91	8.3	C-O, C-N
		287.65	1.91	4.8	O-C-O, C=O
		289.38	1.91	1.0	COOH
	O1s	530.02	2.07		metal oxide (substrate)
		531.71	2.07	8.6	O=C
		533.30	2.07	1.0	O-C
	N1s	398.55	2.17	2.2	NR_3
		400.22	2.17	1.0	HNR^{3+}
	Ca2p3/2	349.06	2.17	1.0	Ca(II)
pristine CNS-Pd	C1s	285.00	1.69	9.0	C-C
		286.28	1.69	3.8	C-O, C-N
		287.57	1.69	2.1	O-C-O, C=O
		288.94	1.69	1.0	COOH
	Pd3d5/2	337.38	1.31	1.0	PdO or Pd(II) coord. COO-
		338.77	1.31	4.0	Pd(II) $PdCl_2$
		340.19	1.31	9.0	Pd(II) coord. amine-like groups
	O1s	530.06	2.08		metal oxide (substrate)
		531.84	2.08	1.3	O=C
		533.33	2.08	1.0	O-C
	Ca2p	346.31	2.11	1.0	Ca(II)
	Cl2p3/2	198.40	2.68	1	$PdCl_2$
	N1s	400.30	2.54	3.6	NR_3
		406.36	2.54	1.0	NO^{3-}
recovered CNS-Pd	C1s	285.00	1.62	7.2	C-C
		286.37	1.62	2.9	C-O, C-N, C-B [1]
		287.41	1.62	1.4	O-C-O, C=O
		288.90	1.62	1.0	COOH
	Pd3d5/2	335.46	1.41	2.5	Pd(0)
		338.20	1.41	1.0	Pd(II) coord. halogenated organic molecules or Pd(0)/C
	O1s	530.05	2.19		metal oxide (substrate)
		531.98	2.19	1.0	O=C
		533.51	2.19	1.0	O-C
	N1s	399.95	2.64	12.0	NR_3
		402.89	2.64	1.0	Noxidized (CNS impurities)
	Br3d5/2	68.80	2.57	1.0	Br-C
	B1s	189.86	2.33	1.0	B-C [1]

References

1. Liu, X.; Wang, Y.; Dong, L.; Chen, X.; Xin, G.; Zhang, Y.; Zang, J. One-step synthesis of shell/core structural boron and nitrogen co-doped graphitic carbon/nanodiamond as efficient electrocatalyst for the oxygen reduction reaction in alkaline media. *Electrochim. Acta* **2016**, *194*, 161–167, doi:10.1016/j.electacta.2016.02.002.
